# Notes for authors

**DOI:** 10.1155/S1463924687000038

**Published:** 1987


addresses used in the electronic interface are not available, the address buffering and decoding logic (figure 4) must be modified to decode an available memory address.

The hardcopy of the date line 599–600 (BASIC program, table 3) will have to be modified to correspond to the syntax of the computer. For graphical output a printer-specific subroutine will have to be written.

### Acknowledgements

Financial support for this project by the Robert A. Welch Foundation of Houston, Texas, and Texas A & M University's Center for Energy and Mineral Resources is gratefully acknowledged.

### References

1. Mertz, W., *Science*, **213** (1981), 1332.
2. Brinckman, F. E. and Bellama, J. M. (Eds), Organometals and organometalloids: occurrence and fate in the environment, *ACS Symposium Series, 82* (1978).
3. Brinckman, R. E., Blair, W. R., Jewett, K. L. and Iverson, W. P., *Journal of Chromatographic Science*, **15** (1977), 493.
4. Koizumi, H., Hadeishi, T. and McLaughlin, R., *Analytical Chemistry*, **50** (1978), 1700.
5. Stockton, R. A. and Irgolic, K. J., *International Journal of Environmental Analytical Chemistry*, **6** (1979), 313.
6. Irgolic, K. J., *Speciation of Arsenic Compounds in Water Supplies*, Report 1982, EPA/600/1–82/010, Order No. PB 82-2578717, 125 pp.; *Chemical Abstracts*, **98**, 113378 (1983).
7. Vickrey, T. M., Buren, M. S. and Howell, H. E., *Analytical Letters*, **A11** (1978), 1075.
8. Vickrey, T. M. and Eue, W., *Journal of Automatic Chemistry*, **1** (1979), 198.
9. Vickrey, T. M., Howell, H. E. and Paradise, M. T., *Analytical Chemistry*, **51** (1979), 1880.
10. Vickrey, T. M., Howell, H. E., Harrison, G. V. and Ramelow, G. J., *Analytical Chemistry*, **52** (1980), 1743.
11. Jinno, K., Tsuchida, S., Nakannishi, S., Hirata, Y. and Fujimoto, C., *Applied Spectroscopy*, **37** (1983), 258.
12. Irgolic, K. J., Stockton, R. A., Chakraborti, D. and Beyer, W., *Spectrochimica Acta*, **38B** (1983), 437.
13. Brinckman, F. E., Jewett, K. L., Iverson, W. P., Irgolic, K. J., Ehrdt, K.C. and Stockton, R. A., *Journal of Chromatography*, **191** (1980), 31.
14. Brown, L., Haswell, S. J., Rhead, M. M., O'Neill, P. and Bancroft, K. C. C., *Analyst*, **108** (1983), 1511.
15. Haswell, S. J., O'Neill, P. and Bancroft, K. C. C., Proc. Int. Conf. Heavy Metals in the Environment, Sept. 6–9, 1983, Heidelberg, Germany; published by CEP Consultants Ltd, 26 Albany Street, Edinburgh EH1 3QH, England. **2** (1983), 1215.
16. Haswell, S. J., Ph.D. Dissertation, Plymouth Polytechnic, Plymouth, England, 1983.

### NOTES FOR AUTHORS

*Journal of Automatic Chemistry incorporating Journal of Clinical Laboratory Automation* covers all aspects of automation and mechanization in analytical, clinical and industrial environments. The *Journal* publishes original research papers; short communications on innovations, techniques and instrumentation, or current research in progress; reports on recent commercial developments; and meeting reports, book reviews and information on forthcoming events. All research papers are refereed.

### Manuscripts

Two copies of articles should be submitted. All articles should be typed in double spacing with ample margins, on one side of the paper only. The following items should be sent: (1) a title-page including a brief and informative title, avoiding the word 'new' and its synonyms; a full list of authors with their affiliations and full addresses; (2) an abstract of about 250 words; (3) the main text; (4) appendices (if any); (5) references; (6) tables, each table on a separate sheet and accompanied by a caption; (7) illustrations (diagrams, drawings and photographs) numbered in a single sequence from 1 upwards and with the author's name on the back of every illustration; captions to illustrations should be typed on a separate sheet. Papers are accepted for publication on condition that they have been submitted only to this *Journal*.

### References

References should be indicated in the text by numbers following the author's name, i.e. Skeggs [6]. In the reference section they should be arranged thus:

to a journal

Manks, D. P., *Journal of Automatic Chemistry*, **3** (1981), 119.

to a book

Malmstadt, H. V., in *Topics in Automatic Chemistry*, Ed. Stockwell, P. B. and Foreman, J. K. (Horwood, Chichester, 1978), p. 68.

### Illustrations

Original copies of diagrams and drawings should be supplied, and should be drawn to be suitable for reduction to the page or column width of the *Journal*, i.e. to 85 mm or 179 mm, with special attention to lettering size. Photographs may be sent as glossy prints or as negatives.

### Proofs and offprints

The principal or corresponding author will be sent proofs for checking and will receive 50 offprints free of charge. Additional offprints may be ordered on a form which accompanies the proofs.

*Manuscripts should be sent to either Dr P. B. Stockwell or Ms M. R. Stewart, see inside front cover.*

